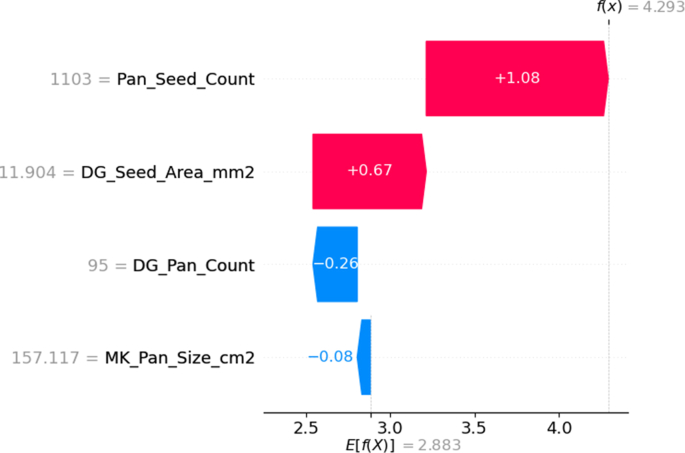# Deep learning for sorghum yield forecasting using uncrewed aerial systems and lab-derived imagery

**DOI:** 10.1016/j.plaphe.2025.100133

**Published:** 2025-12-12

**Authors:** Md. Abdullah Al Bari, Aliva Bakshi, Jahid Chowdhury Choton, Swaraj Pramanik, Trevor D. Witt, Doina Caragea, Scott Bean, S.V. Krishna Jagadish, Terry Felderhoff

**Affiliations:** a Department of Agronomy, Kansas State University, Manhattan, KS, 66506, USA; b Department of Computer Science, Kansas State University, Manhattan, KS, 66506, USA; c Department of Entomology, Kansas State University, Manhattan, KS, 66506, USA; d Center for Grain and Animal Health Research, ARS-USDA, Manhattan, KS, 66506, USA; e Department of Plant and Soil Science, Texas Tech University, Lubbock, TX, 79409, USA; f Department of Plant and Environmental Sciences, New Mexico State University, Las Cruces, NM, 88003, USA

**Keywords:** Deep Learning, Computer Vision, YOLO, Faster-RCNN, Unmanned Aerial Systems (UAS) Imagery, Extracting Yield Features, Sorghum Yield Prediction using Machine Learning

## Abstract

The AI revolution, advanced Graphics Processing Units (GPUs), and open-source platforms have enabled Machine Learning (ML) and Deep Learning (DL) algorithms to rapidly and accurately extract phenotypic features from imagery. Such advancements have led to phenotypic digitization and made rapid yield forecasting possible. Yield predictions are critical to assess the merit of genotypes to propel cultivar development. This trial followed a three-replicated Randomized Complete Block Design (RCBD) with 36 diverse sorghum genotypes in 2023 at Ashland Bottoms, Kansas. The field images were captured 6 m above using a DJI M300 drone at 90° nadir and 45° oblique angles. This research trained YOLO and Faster R-CNN (Detectron2) models to harness yield attributes from UAS field and lab images. The YOLO models outperformed the Faster R-CNN in detecting sorghum panicles, achieving a mean average precision at 50 % IoU (mAP@0.50) scores of 0.92–0.98, compared to 0.61–0.89 for Faster R-CNN. Panicle detection from field imagery showed a linear correlation of 0.86 with ground truth field panicle counts. Lab imagery analyses measured panicle area, seed counts, and seed area with correlation coefficients of 0.79, 0.94, and 0.25 with respective ground truth observations. Support Vector Regression (SVR), Random Forest Regression (RFR), and Decision Tree Regression (DTR) were used to predict yield with correlation coefficients of 0.74, 0.71, and 0.78, respectively, and SHapley Additive exPlanation (SHAP) analysis revealed panicle seed count as the primary driver of yield prediction. We observed YOLO models are well-suited for extracting yield-predictive features from pertinent images. Such features can then be incorporated into ML regression models to predict yield *per se* performance with greater accuracy. The GitHub link is provided in the Data availability section.

## 1 Introduction

The growing global population drives food production increase of 60–70 % by 2050 to meet the projected population growth of 56 % [1]. Scientists, including plant breeders, work toward achieving food and nutritional security. Sorghum, a staple in arid and semi-arid regions, is crucial for nutrition, feedstock, and bioethanol production. Sorghum, as a low-input, drought-tolerant, phenotypically plastic, and genetically diverse crop, offers the potential for yield and trait improvement. Its resilience to low input, water scarcity, and other stresses makes it an increasingly important crop [2]. The renewed interest in sorghum has driven efforts to accelerate the development of cultivars for local and global food security. Researchers focus on enhancing critical yield-predictive features through genetic dissection, conventional breeding, and extensive yield trials across locations and years. Sorghum yield is the primary trait of interest for breeders, agronomists, and growers; early, precise, and scalable preharvest yield predictions are crucial but traditionally rely on labor-intensive, time-consuming manual harvesting [3].

Plant breeders need accelerated and accurate tools to record plant measurements in extensive trials for efficient cultivar development. Advancements in remote sensing, high-throughput phenotyping (HTP), and Uncrewed Aerial Systems (UAS) offer innovative approaches to phenotype crop plants through high-resolution imagery and videos. Such UAS-derived phenotyping has shown a high correlation with ground truth plant observations. However, the abundance of data captured in large breeding programs presents a challenge in processing and deriving meaningful observations for downstream analysis [4]. Image processing methods are critical to effectively leverage the advantages of these vast imagery resources. Deep Learning (DL) has revolutionized Computer Vision (CV) partly due to the advancement and integration of Graphics Processing Units (GPUs), surpassing traditional image processing in terms of their ability to analyze massive and complex datasets to detect objects and efficiently extract meaningful features [5].

Deep Learning, a branch of Machine Learning (ML), leverages deep neural networks such as Convolutional Neural Networks (CNN), Recurrent Neural Networks (RNN), transformer networks, and so on to discover intricate structures in large datasets [6]. Deep Learning excels in processing large datasets by automatically learning a variety of features at multiple levels of abstraction during model training. Such capabilities make DL [7] particularly suitable for complex tasks like plant phenotyping, where efficient feature extraction from high-dimensional data is essential. With the advancement of computational speed, GPU parallel computing, and overall efficiency, pre-trained open-source DL models/networks are now available after training on extensive datasets [8]. Such open-source models can be fine-tuned to perform specific object detection or other relevant computer vision tasks through transfer learning [9]. This approach circumvents the need to train models from scratch, a computationally intensive and time-consuming process.

Efforts to estimate sorghum yield have leveraged images from mobile applications, focusing on panicle counts and the number of grains per panicle [10], as well as drone-based panicle detection pipelines [11]. Building on these approaches, our work for sorghum yield estimation is taking a step forward, by using UAS-captured red, green, blue (RGB) imagery from the field to develop a training dataset, unlike James et al. [11], where manual hand-held machines were used to create a training dataset.

The You Only Look Once (YOLO) [12] platform is widely used in agriculture for detecting crop diseases, pests, and mapping weeds [13,14]. It has also been applied with greater accuracy in detecting sorghum panicles [11,15]. Detectron2 is a comprehensive platform for object detection, segmentation, and other visual recognition tasks, offering implementations of various state-of-the-art models, including Faster R-CNN [16]. This model integrates a region proposal network and a detection network that share convolutional features to streamline the region proposal process [17,18]. This integration results in a highly efficient, accurate, and unified framework for object detection [19].

Forecasting complex Ag attributes often requires specialized ML algorithms, as traditional DL methods may fall short due to their need for extensive data and high computational demands. Support Vector Regression (SVR) has proven effective among various regression models. This algorithm applies the Support Vector Machines (SVM) principles and statistical learning theory [20,21] to minimize prediction errors. Similarly, Random Forest Regression (RFR), an ensemble approach that utilizes multiple decision trees trained on diverse subsets of data, has proven to be effective for regression tasks. This method leverages a randomly sampled subset of features [22], enhancing its ability to effectively predict complex traits such as yield. Additionally, Decision Tree Regression (DTR) [23] can be used for regression tasks. This algorithm builds a single tree that recursively partitions the data into subsets based on feature values, minimizing error [24].

Accurate prediction can significantly assist crop breeding, agronomy, and other plant sciences domains, including enhanced decision making [25] by providing high-quality data promptly. Such efforts enable the quick evaluation of extensive breeding entries, increase selection accuracy, shorten breeding cycles, and efficiently allocate resources toward the most promising genotypes [10,26].

We hypothesized that DL algorithms can effectively extract yield-predictive features from appropriate imagery for sorghum yield forecasting. This research covers a comprehensive workflow from UAS, manual image acquisition, and image processing to deploying DL frameworks to extract sorghum yield-predictive features such as the number of panicles, panicle area, seed counts per panicle, and seed area. These features were then used as input for ML regression models to forecast the yield performance across sorghum genotypes.

Initially, we cropped and labeled UAS imagery and applied YOLO and Faster R-CNN models to detect and count sorghum panicles from field imagery. Subsequently, panicle sizes were extracted using a fine-tuned YOLO model, SAM 2 mask segmentation, and also directly from LabelMe [27] bounding boxes. In the following experiments, we employed a fine-tuned YOLO model to count panicle seeds and used SAM 2 [28] masking to measure seed area. By integrating these DL-driven yield-predictive features into machine learning regressions, we successfully predicted yield with improved accuracy, demonstrating the potential of DL in precision agriculture and crop improvement. SHAP (SHapley Additive exPlanations) [29] values were calculated to explain the interpretability of our ML models. The summary workflow is presented in Fig. 5.

## 2 Materials and methods

The field trial consisted of 36 US Sorghum lines, laid out in a Randomized Complete Block Design (RCBD) with three replicates at Ashland Bottoms, Kansas, with field co-ordinates 39°8′20.49″N, 96°38′21.54″W (39.1384139, −96.6393167) in 2023. The experimental unit followed 18.5-foot-long 4-row plots, with a row spacing of 30 inches and an alley length of 3 feet. The trial was harvested on November 13, 2023.

### 2.1 Field image acquisition

We acquired uncrewed aerial systems (UAS) images from September 25 to 27, 2023, from 2 p.m. to 5 p.m. Using a live video feed, we centered the plot and captured images. The imaging was performed using a DJI M300 (DJI, Shenzhen, China) with a ZenmuseP1 sensor equipped with a 35 mm focal length lens (Fig. 1). A UAV of this size is necessary to carry a full-frame camera. Smaller UAVs could be used to collect imagery for the data pipeline described in this manuscript. However, smaller UAVs would necessarily mean that the sensors would be smaller. This would change the camera specifications, which include the photoreceptor size, megapixels, and focal length. All of these parameters could influence the accuracy of the data pipeline.

**Fig. 1:**
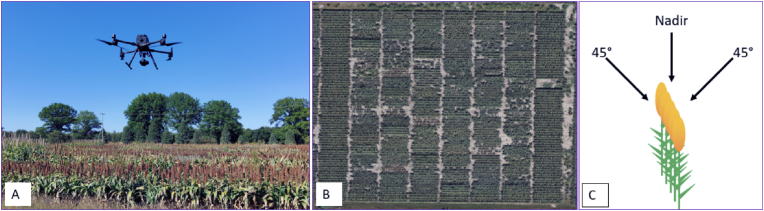
Image capturing and aerial view of the trial: A. DJI M300 Drone during image acquisition. B. Aerial view of the Ideotype trial at Ashland Bottom, 2023. C. Cartoon showing three captured angels of field imagery.

During image capture, the flight Above Ground Level (AGL) was 6 m (m) because that was the lowest altitude we could go without severely disturbing the plants (blowing them around), and the shutter speed was 1/500 seconds. The exposure setting was ISO 200 and aperture 5.6 for all flights to achieve a 0.0 exposure. We manually operated this flight. The camera was focused on the center of the plot for each image. For the training dataset, we captured images of each line from three angles: Nadir (90° from top), 45° facing north, and 45° facing south. We imaged from three angles because of the shadow variability in each view angle and the uncertainty regarding which view would potentially perform better. Also, we hypothesize that multiple view images can improve the robustness of such detection models. The drone was paused for each image. We captured high-resolution Facing South (FS) 36 images first, followed by 36 Nadir (ND) images, and then 36 Facing North (FN) images. Nadir Ground Sample Distance (GSD) was 0.08 cm/pixel, oblique 0.1 cm/pixel. The white balance was kept sunny. Images were captured with clear skies and low wind (<10 mph). Three images per plot were taken in a single replication (Fig. S1).

### 2.2 Lab data acquisition

We randomly sampled and manually harvested four panicles from each plot and set up a stage in the greenhouse to capture images. We recorded the panicle length and width (cm), and their multiplied product, the panicle area (cm^2^), was used as the ground truth panicle area. The stage featured a black cloth backdrop and a measuring scale alongside the panicles. The panicle images were captured from both front and back views. After imaging, the panicles were threshed and grains were spread evenly on a piece of black cloth for further imaging. We used a stand and marked position on the floor and on the stand to ensure fixed camera position during image capture. All images were taken using a Canon EOS REBEL SL1 camera with a 37 mm focal length, a resolution of 72 dpi (both horizontal and vertical), and a 24-bit color depth. The distance between the lens and the objects was 38 inches (Fig. 2). Machine counts were recorded for each panicle after threshing and used as ground truth for the number of seeds per panicle.

**Fig. 2:**
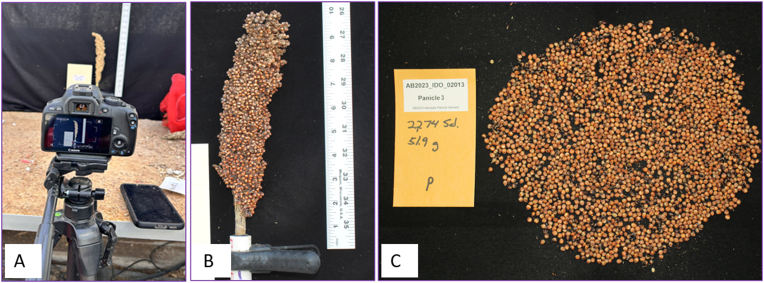
Lab image capture setup: A. RGB camera to capture harvested panicles. B. Image of captured panicle. C. Captured image of the spread seeds.

### 2.3 Image cropping and annotation

The captured raw images were large, with an approximate dimension of 8192 pixels (width) x 5460 pixels (height). These images were cropped to 1280 x 1280 pixels and then resized to 640 x 640 pixels (Fig. 3).

**Fig. 3:**
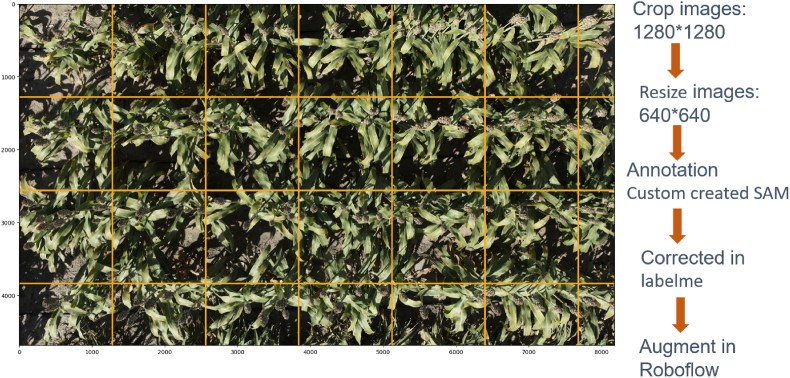
Custom image processing workflow: left panel shows a large image (8192 pixels × 5460 pixels) with 1280 x 1280 grids (orange grid) as an example of input image to workflow; right panel depicts the steps of the workflow, as follows: image were first cropped at 1280 x 1280 resolution, resized to 640 x 640, annotated using a custom SAM 2, annotations were corrected using LabelMe, and finally image augmentations were generated using Roboflow. The final imagery dataset generated with this workflow is used to train YOLO and Faster R-CNN models.

This allowed the image size to be appropriate for model training in YOLO and Faster R-CNN. We fine-tuned the Segment Anything Model 2 (SAM 2) [28] and generated custom annotation tools based on H-ViT model architecture to segment masks around sorghum panicles quickly (Fig. 4). Then, we generated bounding boxes for annotation around the mask. The process resulted in a few incorrect bounding boxes due to overlapping, occlusion, or hiding of panicles.

**Fig. 4:**
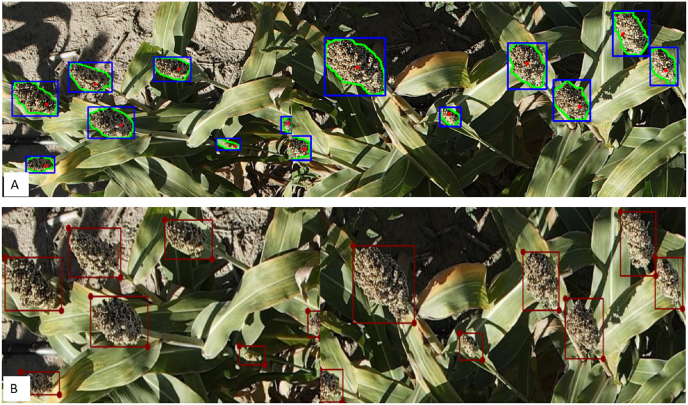
Annotation and Correction: A. Annotated images using fine-tuned Segment Anything Model 2 (SAM 2). B. Correcting annotation using LabelMe.

**Fig. 5:**
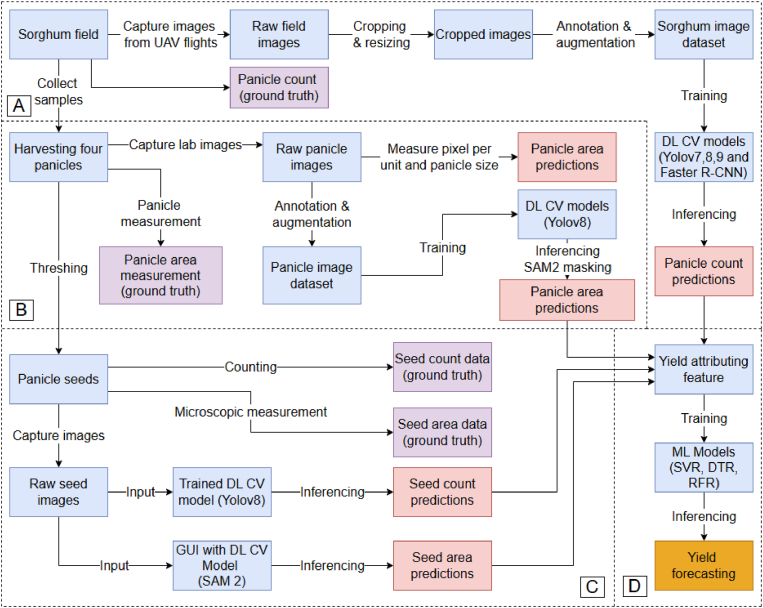
A flow diagram illustrating the steps followed to go from imagery to sorghum yield forecasting: A. Aerial UAS imagery is processed to predict panicle counts. B. Completely random sample panicles are harvested and annotated; panicle area is calculated by multiplying extracted length and width (cm). C. Panicles are threshed, and seeds are machine counted to use as ground truth. A fine-tuned YOLOv8 model is used to detect seeds within panicles and in spread arrangements. SAM 2 is employed to measure individual seed area. D. Outputs from all three experiments are integrated into ML models to forecast sorghum yield.

We used LabelMe [27] to open previously annotated panicle heads (Fig. 4) and manually correct incorrect bounding boxes. This process is faster than manual annotation since a single click generates a segmentation mask, avoiding the need to draw bounding boxes for each panicle.

We developed four types of datasets for panicle detection based on view angles: ND, FN, FS, and All view angles (Table S1). Nadir View started with 303 original images, which, after preprocessing and augmentation, expanded to 841 images, distributed as 807 for training, 17 for validation, and 17 for testing. The comprehensive All view Angles dataset was created by combining images from ND, FN, and FS categories, totaling 1627 images–1569 for training, 29 for validation, and 29 for testing, following a 90:5:5 split. We also experimented with an 80:10:10 split, with relatively less performance in the prediction; therefore, this experiment was not included. The training, validation, and testing subsets are crucial for developing, fine-tuning, and evaluating the model's performance. This dataset was designed to enhance the robustness and accuracy of panicle detection. All images are auto-oriented, resized to 640 × 640 pixels, and converted to grayscale. We included grayscale to prioritize object detection. For further details on other view angles, please refer to Table S1. All preprocessing and augmentation were conducted in Roboflow [30].

### 2.4 DL models for object detection

An object detection model usually consists of a backbone, a neck, and a head. A convolutional neural network (CNN) pre-trained on the ImageNet dataset [31] can form the backbone and generate feature representations. The neck usually consists of more CNN layers and is used for feature enhancement. The head is responsible for identifying objects by predicting bounding boxes around objects and classifying the objects [32]. We used object detection models, such as Faster R-CNN, implemented using the Detectron2 framework, and three versions of YOLO, available through the Ultralytics Python library [33].

Faster R-CNN is an advanced version of region-based Convolutional Neural Networks (CNNs), initially proposed by Girshick et al. [16] and further developed by Ren et al. [18]. It consists of two neural networks: the Region Proposal Network (RPN) and the Fast R-CNN detector, which work together to identify and classify objects in images. The process starts with an input image passing through a convolutional neural network, such as VGG16, ResNet50, or ResNet101, which produces feature maps used to identify potential object locations as regions of interest (RoIs). The RoIs are subsequently refined and classified by the Fast R-CNN network. The final model is a highly accurate object detector that predicts bounding boxes, object locations, dimensions, class labels, and confidence scores.

YOLO is a one-stage object detector that simultaneously localizes and classifies objects by framing detection as a regression task. YOLO processes full images in a single step, directly predicting bounding boxes and class probabilities. Each bounding box contains the center coordinates (x, y), the width (w), and the height (h). This orientation enables end-to-end optimization for detection performance, significantly enhancing processing speed. Also, Non-Maximum Suppression (NMS) was applied to refine predictions to eliminate redundant bounding boxes, ensuring the most probable detections [34]. We leveraged YOLO models using the Ultralytics Python library [33].

We deployed YOLOv7, YOLOv8, and YOLOv9 models to compare their strengths in speed, accuracy, and computational efficiency. YOLOv7's original evaluation showed that it excelled in real-time object detection, outperforming both transformer-based (SWINL Cascade-Mask R-CNN) and convolutional-based (ConvNeXt-XL Cascade-Mask R-CNN) detectors, as well as previous YOLO versions [35]. YOLOv8 was built on YOLOv5 with improved speed and robustness, enhancing detection capabilities, especially for small objects [36], while YOLOv9 introduced Programmable Gradient Information (PGI) and a lightweight Generalized Efficient Layer Aggregation Network (GELAN), which mitigated data loss and further improved accuracy and computational cost, surpassing earlier models like YOLOv7 and the larger YOLOv8-X [37].

### 2.5 Field panicle detection and counting

We processed the large UAS captured field imagery by cropping, resizing, annotating, and augmenting it to generate training datasets consisting of 841, 614, 547, and 1627 (Table S1) images for ND, FN, FS, and All View angle images, respectively. We leveraged advanced object detection frameworks, each optimized through careful tuning of model parameters to maximize performance to detect and count panicles from the field imagery.

We began with the Faster R-CNN model implemented in Detectron2. The model used a ResNet-50 backbone and Feature Pyramid Network (FPN). The model was trained for 300 iterations with a base learning rate of 0.007, and checkpoints were saved every 150 iterations. The training configuration processed two images per batch, with Region of Interest (ROI) heads handling 10,240 regions per image and an Intersection over Union (IoU) threshold set at 0.7. The Region Proposal Network was set to manage up to 2500 proposals post non-maximum suppression. The best-performing model weights were saved and later used to infer and count panicles from the full-resolution field images.

We also trained YOLOv7 models using the same datasets across three view angles and their combinations. Training was performed with pretrained base weights for 100 epochs, using a batch size of 14 and an input resolution of 640 × 640 pixels. A maximum of 400 panicles was allowed per image during inference. The best checkpoint was selected based on validation performance, and inference was carried out with a low confidence threshold adjusted to maximize detection coverage.

YOLOv8 models were trained with same datasets with small base weight initialization, with a configuration designed for 100 epochs, a batch size of 8, and a learning rate of 0.001. We also maintained the same object detection and image size parameters as YOLOv7.

Finally, YOLOv9 models were initialized with the efficient variant of the architecture, with training configured for 100 epochs at a learning rate of 0.01. The momentum and weight decay were set to 0.937 and 0.0005, respectively, to balance convergence speed and generalization. Detection was performed using the final optimized model's weight to ensure high accuracy during inference.

Across all models, the training and inference configurations were carefully selected to reflect the operational demands of large-scale field phenotyping, ensuring robust detection performance across different view angles. After panicle inferences, we obtained digital panicle counts for one replication. These counts were then compared with ground truth panicle counts to calculate the slope and intercept of a linear regression model. Using the resulting regression equation, we predicted panicle counts for the remaining two replications by applying the estimated slope and intercept to the trained model.

### 2.6 Measuring panicle area

We annotated harvested laboratory panicle images with rectangular bounding boxes in LabelMe tool [27]. From each annotation, we extracted the pixel length and width of the panicle using the bounding box given by (*x*_1_, *y*_1_) and (*x*_2_, *y*_2_) coordinates. The width and height in pixels were computed as the absolute differences of the x and y (|*x*_2_ − *x*_1_| and |*y*_2_ − *y*_1_|) coordinates, respectively.

From each plot, four panicles were harvested and imaged. A total of 324 images were used to train a YOLOv8 model, and the best-performing version was saved during training. This fine-tuned model was subsequently used to infer on a test set of 108 previously unseen lab images. Panicle length and width were measured from the predicted bounding boxes. Each bounding box contains the center coordinates (x, y), the width (w), and the height (h). By iterating through the bounding box outputs, we retrieved the width and height pixels of each detected panicle. Additionally, SAM 2 mask segmentation was applied to the YOLO-predicted bounding boxes to extract the panicle area in pixels size within each box.

We determined the number of pixels per unit of physical length (per cm/inch) using panicle images with a reference ruler (Fig. 2. B). The pixel dimensions were then converted into physical panicle length and width measurements using the determined pixel counts per cm/inch scale; panicle length and width in cm were then multiplied to obtain panicle area (cm^2^). SAM 2 mask segmentation derived pixels of the panicle area were converted to mask panicle area (cm^2^) by dividing pixels per cm^2^ area. The manually recorded panicle length (cm) and width (cm) were multiplied and used as the ground truth panicle area for this experiment, as mentioned in the lab data acquisition segment.

We also evaluated the best weights saved from the YOLOv8 model fine-tuned on the FN field dataset by making predictions on the lab-captured panicle images, after resizing to 640 x 640. We hypothesized that the FN view would give better detection in the lab panicles as the FN view had more exposed panicles and better lighting conditions. In this instance, the pixel measurements were not converted into physical units because image resizing altered the scale, making the conversion inaccurate; therefore, the panicle dimensions were reported in pixels. Panicle area was averaged across four panicles per plot, except the digitally obtained mask panicle area, which had a single observation per plot.

### 2.7 Counting seeds

Spread seed count data were available for a single replication across four panicles on 144 images. Since we did not capture spread threshed imagery for replications two and three, we predicted spread count seed using a linear regression model after training on the first set of replication data. In this model, ground truth seed count was used as the independent variable and spread seed count as the dependent (response) variable. We applied the resulting regression equation using the appropriate slope coefficient and intercept in the original 144 observations to predict spread seed count for replication two and three. Overall extracted seed counting are averaged across four sampled panicles per plot. We captured forward and backward images of four harvested intact panicles from each experimental plot, serving as a paired dataset. Additionally, after threshing each panicle, we imaged the spread grains. To estimate seed count, we applied a fine-tuned detection-based seed counting model [38] to detect and count seeds in the forward and backward images of the panicles (Pan_Seed_Count) (Fig. S6 A & B). Since some seeds were occluded and not directly visible, we also employed a regression-based seed counting approach (Reg_Seed_Count), using ResNet50-based regression [39] as suggested by Bakshi et al. [38]. We also estimated seed counts from threshed grain described as spread seed count (Spr_Seed_Count) images (Fig. S6 C) using a YOLOv8 model, based on images where seeds were spread on a piece of black cloth. The machine count of the threshed seeds (Mach_Seed_Count) was used as the ground truth to evaluate the accuracy of the other seed counting methods.

### 2.8 Measuring seed area

We developed a graphical user interface (GUI) leveraging the Python library Matplotlib to facilitate interactive seed area calculations. The interface is powered by SAM 2 with H-ViT model architecture [28]. We developed an image viewer class to visualize and interact with images. Users can click on sorghum seeds to initiate object masking, highlighting the seed surface with displayed contours. For each contour, the maximum Euclidean distance, representing the diameter, is calculated between any two points on the contour. This distance is typically measured in pixels.

Sorghum seeds are elliptical in appearance, with one long and one short axis, alternatively, the length and width of the seed. To estimate the length and width of each seed, we utilized the bounding box coordinates generated by the SAM 2 masking. The bounding box dimensions were calculated as follows: the horizontal extent was determined by subtracting the leftmost edge (x1) from the rightmost edge (x2) (subtracting lower values from the higher values), and the vertical extent was determined by subtracting the bottommost edge (y1) from the topmost edge (y2). Since seed orientation was random in the images, the long axis could align with either the x-axis or y-axis, varying from seed to seed. The greater of these two extents was considered the seed length, while the smaller was classified as the seed width.

To convert the pixel distance to standard measuring units, we first convert pixels to millimeters (mm) using the recorded pixel-per-millimeter values from the spread seed images with labels of known dimensions. The conversion is defined by:

$$\text{Distance }\left(\text{mm}\right)=\frac{\text{Distance }\left(\text{in pixels}\right)}{\text{pixels per mm}}$$

Finally, the seed area in square millimeters (mm^2^) is calculated based on the length and width using the area formula for an ellipse:

$$\text{Digital Area }=\pi \left(\frac{\text{length}}{2}\right)\times \left(\frac{\text{width}}{2}\right)$$

We randomly pointed and clicked 10 to 20 seeds, obtained the seed area, and calculated the means. The graphical interface enables the visualization of seed contours and allows quick and efficient measurement and calculation of seed areas. This platform lays the foundation for capacity building for extensive and rigorous phenotypic evaluation (Fig. S2).

As ground truth, we randomly collected 10 seeds from each of the 108 plots and measured the seed length and width using a Keyence VX-6000 digital microscope (Fig. S2). Image analysis tools were used to measure the length and width of the individual kernels (mm). The area of each kernel was measured by tracing the outline of each kernel. Individual kernels were also analyzed using the Single Kernel Characterization System (SKCS) [40] to measure the seed diameter (thickness) (mm) and the seed weight (mg) of the same 10 seeds.

### 2.9 Yield forecasting

We used Support Vector Regression (SVR) [20], Random Forest Regression (RFR) [22], and Decision Tree Regression (DTR) [23], to forecast sorghum yield (ton/ha) using the Scikit-learn Python library [41]. Yield was the response variable (y), while independent variables (X) included digitally obtained yield-predictive features: panicle count (*DG_Pan_Count*), SAM 2 mask derived panicle area (cm^2^) (*Mask_Pan_Area_cm*^*2*^), panicle seed count (*Pan_Seed_Count*), and digital seed area (*Digital_Area_mm*^*2*^). We randomly partitioned the dataset of 108 entries into training and testing sets using an 80-20 split with a fixed random seed to ensure reproducibility. The analysis was performed using Scikit-learn, which does not have explicit splitting for validation during model training; therefore, a validation set was used in this study.

We digitally extracted 11 yield-predictive features, each exhibiting a different degree of correlation with the yield, ranging from 0.05 to 0.54∗∗∗ (Fig. S7). These features also showed strong inter-correlations, indicating possible collinearity. To address this, we conducted Principal Component Analysis (PCA) to assess the relative contribution of each feature to yield variability. Based on the PCA results and correlation analysis, we selected a subset of features for the ML regression models to minimize redundancy and avoid collinearity.

The PCA revealed that SAM 2 mask derived panicle area (Mask_Pan_Area_cm^2^) explained 42 % of the yield variability. This was closely followed by the panicle area (B_Box_Pan_Size_cm^2^) derived from LabelMe bounding boxes, contributing 39 % of the variability (Table S4). However, due to their higher mutual correlation (r = 0.73∗∗∗) (Fig. S7), only the SAM 2-derived panicle area was included in the ML models. Similarly, other digitally obtained panicle features contributed 34 % and 33 % of the yield variability (Table S4), and were also excluded from the ML algorithm due to their correlation with SAM 2 mask derived panicle area at 0.70∗∗∗ and 0.43∗∗∗ (Fig. S7). Among seed counting features, the seed count obtained from panicle images (Pan_Seed_Count) was retained into ML models due to its high correlation with yield ton/ha (r = 0.54∗∗∗). The spread seed count (Spread_Seed_Count) and regression seed count (Reg_Seed_Count) were dropped from the predictive model to avoid redundancy. We added digital seed area mm^2^ among the extracted seed area features into the ML predictive models.

The SVR model was initialized with a Radial Basis Function (RBF) kernel–a common choice for nonlinear data. We trained the SVR with *C* = 1.0, representing the regularization parameter, and *ϵ* = 0.1, defining the epsilon-tube within which no penalty is applied in the training loss function for predictions within a distance *ϵ* from the actual values. Other model parameters were set to their default values as defined in the scikit-learn implementation of SVR, including: degree = 3 (used for polynomial kernels), gamma = ‘scale’ (influence of a single training example), coef0 = 0.0 (independent term in the kernel function), tol = 0.001 (convergence threshold), shrinking = True (enables the shrinking heuristic), cache_size = 200 (memory allocation for the kernel cache, in MB), verbose = False (disables training output), and max_iter = -1 (no limit on iterations) [41].

The model fitting process involved multiple iterations over randomly shuffled data splits to ensure robustness and mitigate overfitting. In each iteration, the model was trained on the training set and then used to predict yield on the test set. The mean squared error (MSE) was computed for each prediction to assess the accuracy of the model. This iterative process helped validate the effectiveness of models across different subsets of data. It allowed us to select the iteration with the lowest MSE, representing the model with the best generalization performance on unseen data. These tasks were executed in the testing dataset.

We trained Decision Tree Regression (DTR) and Random Forest Regression (RFR) algorithms using the default hyperparameters in Scikit-learn, following the implementation of SVR mentioned above to predict sorghum yield based on the extracted yield-predictive parameters. The best-performing model was then used to make final predictions on the never-trained 20 % test dataset. SHAP (SHapley Additive exPlanations) values were computed using the shap package to measure the contribution of each feature locally at individual data points and globally for the collective data points. SHAP is a unified framework based on a game-theoretic approach for interpreting predictions of machine learning models [29]. In this study, we calculated a linear model to estimate SHAP values and assess the influence of each feature on model predictions. To capture local interpretability, SHAP values were calculated for each data point, while a beeswarm plot was generated to visualize the overall impact of features across the entire dataset.

### 2.10 Brief overview of experiments

We conducted several intertwined experiments for extracting yield-predictive features for use in ML models to forecast sorghum yield. This flowchart presents a comprehensive pipeline of experiments used in this study by integrating UAS field, manual imagery, DL-based computer vision models for extracting yield-predictive features, and downstream ML models. The experiments are divided into four key modules described in the caption (Fig. 5).

### 2.11 Computational resources used

The YOLO series models were trained on a Dell Precision 7670 workstation with Python 3.11 development environment under the Linux operating system. This system is equipped with a 12th Generation Intel (R) Core (TM) i7-12850HX processor featuring 24 CPUs and a base clock speed of 2.1 GHz, complemented by 65,536 MB of RAM. It has also, an NVIDIA RTX A1000 Laptop Graphics Processing Unit (GPU) to enhance GPU-based tasks. Additionally, computations were performed on an Ubuntu 22.04.4 LTS platform (GNU/Linux 5.15.0–118-generic x86_64), which was outfitted with an NVIDIA RTX A2000 12 GB GPU and CUDA version 12.4. We also used Google Colab to train Faster R-CNN models, leveraging a T4 GPU and a Python 3 environment.

### 2.12 Model assessment and statistics

In the first experiment, which used four types of imagery data (Table S1), we calculated precision-recall and F1 scores to evaluate the performance of the algorithms on the test and validation datasets [8]. True-positive (TP) and false-positive (FP) detections were determined with an IoU threshold of 0.5 (50 %) between the annotated object and predicted bounding boxes. Intersection over Union is the area of overlap between the ground truth bounding boxes and the predicted bounding boxes projected by the trained DL algorithms [42] divided by the total area of the union of the bounding boxes. If the IoU values are greater than or equal to 0.5, they are considered TP, and values less than 0.5 are considered FP [43].

(1) $$\text{Intersection over Union }\left(\text{IoU}\right)=\frac{\text{Area of overlap}}{\text{Area of union}}$$

The precision of the model is the proportion of correctly predicted objects (True Positives) to the total number of predictions (TP + FP). The precision values range from 0 to 1. Precision values are reduced with incorrect positive detection, meaning higher FP predictions or fewer TP predictions. A higher precision means lower FP and higher TP predictions. Precision values are computed with the equation:

(2) $$\text{Precision}=\frac{\text{TP}}{\text{TP}+\text{FP}}$$

Recall is the fraction of correctly detected objects (TP) out of all ground truth objects (TP + FN), and defines how well a model identifies TP predictions. A higher recall implies better TP predictions. The recall values range from 0 to 1 and are computed with the equation:

(3) $$\text{Recall}=\frac{\text{TP}}{\text{TP}+\text{FN}}$$

The F1 score is the harmonic mean between precision and recall [44]. In other words, F1 is a function that captures the tradeoff between Precision and Recall. The best score is 1, and the worst score is 0. It was calculated as:

(4) $$F1=2\times \frac{\text{Precision}\times \text{recall}}{\text{Precision}+\text{recall}}$$

The average precision (AP) for sorghum panicle detection is determined by graphing a precision-recall curve for each test image and computing the area beneath each curve [43]. The AP is then used to find the mean AP (mAP) of the algorithm, which was calculated from an IoU threshold of 0.50 in this study following the equation:

(5) $$\text{mAP}=\frac{\sum_{i=1}^{N}{\text{AP}}_{i}}{N}$$

We also assessed AP@0.50–0.95, the mean average precision calculated at multiple IoU thresholds ranging from 0.50 to 0.95, with a step of 0.05. This metric provides a comprehensive measure of detection performance, penalizing models that predict inaccurate bounding boxes. It is more robust than the mAP@0.50 since it evaluates precision across different levels of IoU strictness.

In the subsequent experiments, we used fined-tuned models and predicted against ground truth observations. In such instances, we measured the Pearson Correlation Coefficient (r).

(6) $$r=\frac{\sum_{i=1}^{N}\left(y_{i}^{\mathrm{Pred}}-\bar{y_{i}^{\mathrm{Pred}}}\right)\left(y_{i}^{GT}-\bar{y_{i}^{GT}}\right)}{\sqrt{\sum_{i=1}^{N}{\left(y_{i}^{\mathrm{Pred}}-\bar{y_{i}^{\mathrm{Pred}}}\right)}^{2}\sum_{i=1}^{N}{\left(y_{i}^{GT}-\bar{y_{i}^{GT}}\right)}^{2}}}$$

where, *r* is the Pearson Correlation Coefficient, $y_{i}^{\mathrm{Pred}}$ represents model predicted values, $\bar{y_{i}^{\mathrm{Pred}}}$ is mean of the predicted values, $y_{i}^{GT}$ refers to actual ground truth observations, and $\bar{y_{i}^{GT}}$ is the mean of the ground truth observation as shown in Equation (6).

Moreover, in all the experiments, we also calculated error metrics, including Mean Absolute Error (MAE), Normalized Error (NE), Mean Squared Error (MSE), and Root Mean Squared Error (RMSE) (Equations (9), (8), (7), (10)) by comparing predictions to the respective ground truth observations to evaluate model performance. Lower values of these error metrics indicate higher model accuracy.

(7) $$MAE=\frac{1}{N}\overset{N}{\underset{i=1}{\sum }}\left|y_{i}^{GT}-y_{i}^{\text{Pred}}\right|$$

(8) $$NE=\frac{MAE}{GroundTruthMean}$$

(9) $$MSE=\frac{1}{N}\overset{N}{\underset{i=1}{\sum }}{\left(y_{i}^{GT}-y_{i}^{\text{Pred}}\right)}^{2}$$

(10) $$RMSE=\sqrt{\frac{1}{N}\overset{N}{\underset{i=1}{\sum }}{\left(y_{i}^{GT}-y_{i}^{\text{Pred}}\right)}^{2}}$$

## 3 Results

### 3.1 Field panicle detection

We observed varying performance across evaluation metrics using the Faster R-CNN, YOLOv7, YOLOv8, and YOLOv9 models. However, we consistently observed better performance when models were trained using ND and All view angle images to detect panicles. YOLO models performed similarly across versions and image view angles, with AP@0.50–0.95 ranging from 0.64 to 0.90 and AP@0.50 between 0.92 and 0.98 (Table 1). YOLOv7 achieved the highest AP values except for FS, with AP@0.50–0.95 of 0.90 and AP@0.50 of 0.98 when trained on ND view images. YOLOv9 closely followed with an AP@0.50–0.95 of 0.81 and an AP@0.50 of 0.97, while YOLOv8 exhibited slightly lower performance. Though accurate at AP@0.50, Faster R-CNN performed inadequately, especially for FN and FS views. ND view training consistently exhibited improved accuracy, emphasizing suitability for sorghum panicle detection. However, All view images provided a more robust, fine-tuned model after training.

**Table 1:** Comparison of Faster R-CNN, YOLOv7, YOLOv8, and YOLOv9 based on AP@0.50–0.95 and AP@0.50 across four view angles.

Model	ND	FN	FS	All View
AP@0.50–0.95				

YOLOv7	**0.90**	0.86	0.86	0.**86**
YOLOv8	0.76	0.64	0.72	0.67
YOLOv9	0.81	0.69	0.72	0.71
Faster R-CNN	0.42	0.48	0.35	0.59

AP@0.50				

YOLOv7	**0.98**	0.94	0.98	0.**93**
YOLOv8	**0.96**	0.95	0.97	0.**92**
YOLOv9	**0.97**	0.97	0.97	0.**94**
Faster R-CNN	0.84	0.62	0.61	0.89

∗ND is Nadir view, FN is Facing North, FS is Facing South, All View combines images from all the view angles; AP@0.50–0.95 refers to the mean average precision at multiple Intersection over the Union (IoU) thresholds ranging from 0.50 to 0.95; AP@0.50 is the average precision at a 50 % IoU threshold.

The F1 score of each YOLO algorithm, as a function of the prediction confidence, is illustrated in Fig. S3 A. The YOLOv7 model achieved the highest F1 score of 0.90 at a confidence threshold of 0.582, while YOLOv8 obtained an F1 score of 0.89 at 0.589. Similarly, YOLOv9 reached an F1 score of 0.90 at a threshold of 0.465. These thresholds represent the optimal balance between precision and recall, ensuring the best detection performance for each model.

The Precision-Recall (PR) curve visually presents the performance of object detection models (Fig. S3 B). YOLOv7, YOLOv8, and YOLOv9 demonstrated greater accuracy in detecting panicles, with mean Average Precision (mAP) scores of 0.93, 0.92, and 0.94, respectively. Since this study focused on single-class object detection, the mAP at an Intersection over Union (IoU) threshold of 0.5 was identical to the class-specific Average Precision (AP) for each algorithm. The results exhibited robust and comparable performance in panicle detection using all three YOLO models, as indicated by the Precision-Recall (PR) curve.

The choice of confidence threshold can significantly influence detection performance. Lower confidence thresholds, such as 0.1, tend to yield more detections but are prone to higher rates of false positives, leading to incorrect labeling of panicle heads. In contrast, higher thresholds, such as 0.6, may produce fewer detections with increased precision but at the expense of more false negatives. In this study, confidence thresholds of 0.1 for YOLOv7 and YOLOv9 produced results closely matching ground truth observations, whereas YOLOv8 exhibited higher inference accuracy at a threshold of 0.6.

The detected panicles across view angles and DL algorithms are illustrated in Fig. S2. Each YOLO algorithm saved its best-performing weights during training, which we used for inference on large field images. The best-trained model was used to make inferences on the large field images. We found greater accuracy in the ND view images.

Fig. 6 illustrates the correlation between the actual ground truth panicle counts and the panicle numbers predicted by the models in the field plots, using fine-tuned models from: A. All view images and B. ND view images. Both the fine-tuned models were used to predict the ND view images. In addition, correlations between different models are also shown.

**Fig. 6:**
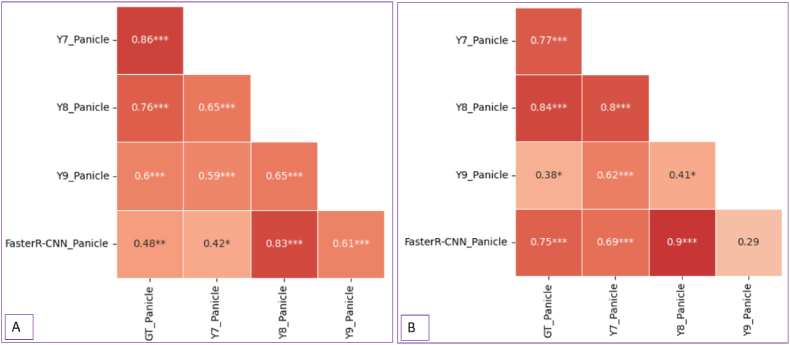
Correlation among ground truth and predicted panicle counts in the field imagery: A. Model trained with All view angle images. B. Model trained with ND view images. Pearson correlations: ∗*P* < 0.05, ∗∗∗*P* < 0.001.

YOLOv7, using the best-trained (fine-tuned) model from All view angle images, predicted very close to the ground truth panicle counts, achieving a correlation of 0.86∗∗∗, followed by YOLOv8 with a correlation of 0.76∗∗∗. We obtained slightly varying outcomes when we trained the model with ND view images; YOLOv8 outperformed YOLOv7, achieving a correlation of 0.84∗∗∗, while YOLOv7 followed a little low with a correlation of 0.77∗∗∗ (Fig. 6 B).

When the best-trained (fine-tuned) model from All view angle images was used to make inferences on ND view images, YOLOv8 achieved the lowest MAE (16.42), followed by YOLOv7 (18.97). In contrast, YOLOv9 (48.39) and Faster R-CNN (47.58) performed considerably worse. A similar trend was observed in the NE, where YOLOv8 yielded the lowest NE (0.11), followed by YOLOv7 (0.13), while YOLOv9 (0.32) and Faster R-CNN (0.31) showed higher errors (Table S2). In the second scenario, where models trained exclusively on ND view images were used for inferences on ND view images, YOLOv8 again outperformed other models with the lowest MAE (14.33), NE (0.09), and RMSE (20.68). YOLOv7 (MAE = 26.89, NE = 0.18, and RMSE = 34.83); YOLOv9 (MAE = 72.94, NE = 0.48, RMSE = 80.7), and Faster R-CNN (MAE = 23.75, NE = 0.16, RMSE = 28.39) performed moderately (Table S2).

We observed consistent advantages of YOLOv8 across scenarios based on performance; YOLOv7 may also serve as a competitive alternative, while YOLOv9 and Faster R-CNN exhibited lower accuracy and reliability in detecting and counting panicles in the field from UAS imagery.

### 3.2 Panicle area

One of the reasons we picked the Ultralytics YOLOv8 model for detecting panicles is its capability to detect objects with high precision while providing detailed bounding box information (coordinates, width, height). The model generates rectangular bounding boxes for each detected panicles, allowing the extraction of the width and height dimensions, which were then multiplied to estimate and predict panicle areas. In addition, the panicle bounding boxes predicted by the trained YOLOv8 model on the lab imagery were also provided prompts to SAM 2 for panicle mask segmentation and area estimation. The best predicted lab panicle area close to the manually measured ground truth area was obtained from SAM 2 based mask segmentation with a correlation coefficient of 0.79∗∗ (Fig. S5). The performance matrices included MAE of 54.46 cm^2^, NE of 0.37, and RMSE of 59.92 cm^2^ (Table S3). We predicted lab panicle area based on YOLOv8 bounding boxes showed a correlation coefficient of 0.59∗∗ (Fig. S5) with the ground truth panicle area. The performance matrices included MAE of 46.31 cm^2^, NE of 0.32, and RMSE of 72.33 cm^2^ (Table S3). The lab panicle dimensions were also estimated using LabelMe annotations. The panicle area estimated from LabelMe bounding boxes is strongly associated with the ground truth panicle area in square centimeters (cm^2^), with a correlation coefficient of 0.71∗∗∗. This suggests that LabelMe annotations are quite reliable for estimating panicle areas. The further performance matrices included MAE of 38.54 cm^2^, NE of 0.26, and RMSE of 57.52 cm^2^ (Table S3). However, SAM 2 based mask segmentation provided the closest estimates to the ground truth panicle area estimation. Significant correlations were also observed between YOLOv8 predicted panicle area in pixels with the ground truth panicle area in cm^2^ (0.37∗∗∗) (Fig. S5).

### 3.3 Seed counting

A trained YOLOv8 model [38] was utilized to count seeds from the front and back views of the harvested panicles. The counts from both views were summed to estimate the total panicle seed count per panicle. We also counted seeds on spread images after threshing the harvested panicles. A strong correlation was observed between the digitally estimated seed counts and the ground truth measurements. The machine counted seeds (*Mach_Seed_Count*) obtained after harvesting and threshing were used as the ground truth for validation. Among the various seed counting methods evaluated, the spread count demonstrated the strongest association with the machine count (*r* = 0.94∗∗∗), followed by the panicle count (*r* = 0.73∗∗∗) and the regression-based count (*r* = 0.56∗∗∗) (Fig. 7).The NE ranging from 0.11 to 0.31 is consistent with the correlation trends, and other error statistics are in Table S3.

**Fig. 7:**
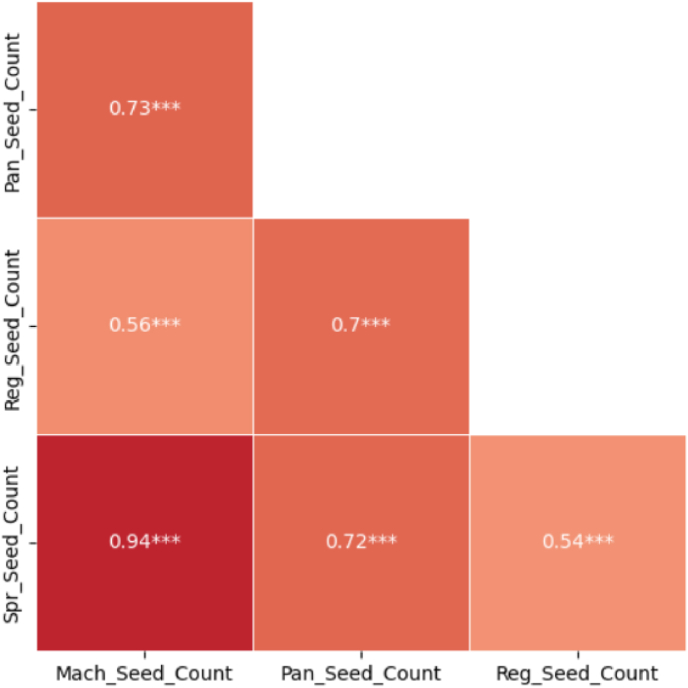
Correlation of machine counted seeds with predicted seeds using a deep learning framework. *Mach_Seed_Count* represents the ground truth machine counts of the harvested and threshed seeds, *Pan_Seed_Count* is the sum of panicle counts from the front and back views of a panicle, *Reg_Seed_Count* is the prediction based on regression count, and *Spr_Seed_Count* is the prediction using trained DL computer vision model on the spread seed images for replication one, and prediction based on trained regression model for replication two and three.∗∗∗ indicates that the Pearson correlation is significant at *P* < 0.001.

### 3.4 Seed area

The seed area was also estimated using a custom-created tool. We calculated the seed area from 10 to 15 random seeds from each genotype. We used another set of 10 randomly selected seeds from the respective genotypes for microscopic seed area (*Microscope_Area_mm*^*2*^) calculation used as ground truth, SKCS kernel measurement (*SKCS_Kernel_dia_mm*), and individual seed weight (*Kernel_weight_mg*). A moderate but significant correlation existed between the digitally estimated seed area and the ground truth measurements. Among the various seed areas calculated, the digitally obtained area is (*r* = 0.25∗∗), associated with the ground truth microscope area estimation (Fig. 8).

**Fig. 8:**
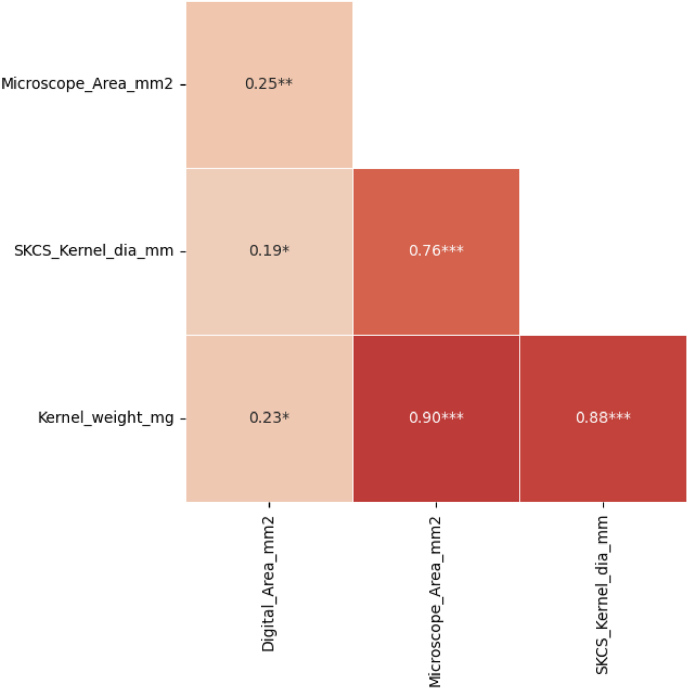
Correlation of digitally obtained seed area with ground truth seed area measurement across 10 random seeds of 108 entries. *Digital_Area_mm*^*2*^ represents digitally obtained seed area, *Microscope_Area_mm*^*2*^ is ground truth area obtained from microscope imagery, *SKCS_Kernel_dia_mm*^*2*^ is Single Kernel ground truth area obtained from microscope imagery, and *Pan_Count* is the sum of panicle counts from the front and back views of a panicle. ∗∗ indicates that the Pearson correlation is significant at *P* < 0.01.

Digitally obtained seed area (*Digital_Area_mm*^*2*^) obtained the least MAE of 2.02 mm^2^, NE of 0.19, and RMSE of 2.71 (Table S3).

### 3.5 Yield forecasting

We compiled digitally obtained yield-predictive features and used them as independent variables in the ML regression models to estimate yield, as detailed in the methodology section. The correlation coefficients for SVM, RFR, and DTR on the test dataset were 0.74∗∗∗, 0.71∗∗∗, and 0.78∗∗∗, respectively (Fig. 9 A, C, E). Among these models, DTR demonstrated the highest reliability as per error statistics with the lowest mean absolute error (MAE = 0.49 ton/ha), normalized error (NE = 0.16), and root mean square error (RMSE = 0.84 ton/ha). SVR provided slightly higher MAE = 1.35 ton/ha, NE = 0.39, and RMSE = 1.66 ton/ha, while RFR had MAE = 0.72 ton/ha, NE = 0.20, and RMSE = 1.02 ton/ha (Table S4). Given the average yield of this trial was 3.43 tons/ha, DTR proved to be the most accurate model in our study for predicting yield. Consistent with the error term, the highest correlation between the predicted and observed yield was obtained from DRT, as mentioned earlier.

**Fig. 9:**
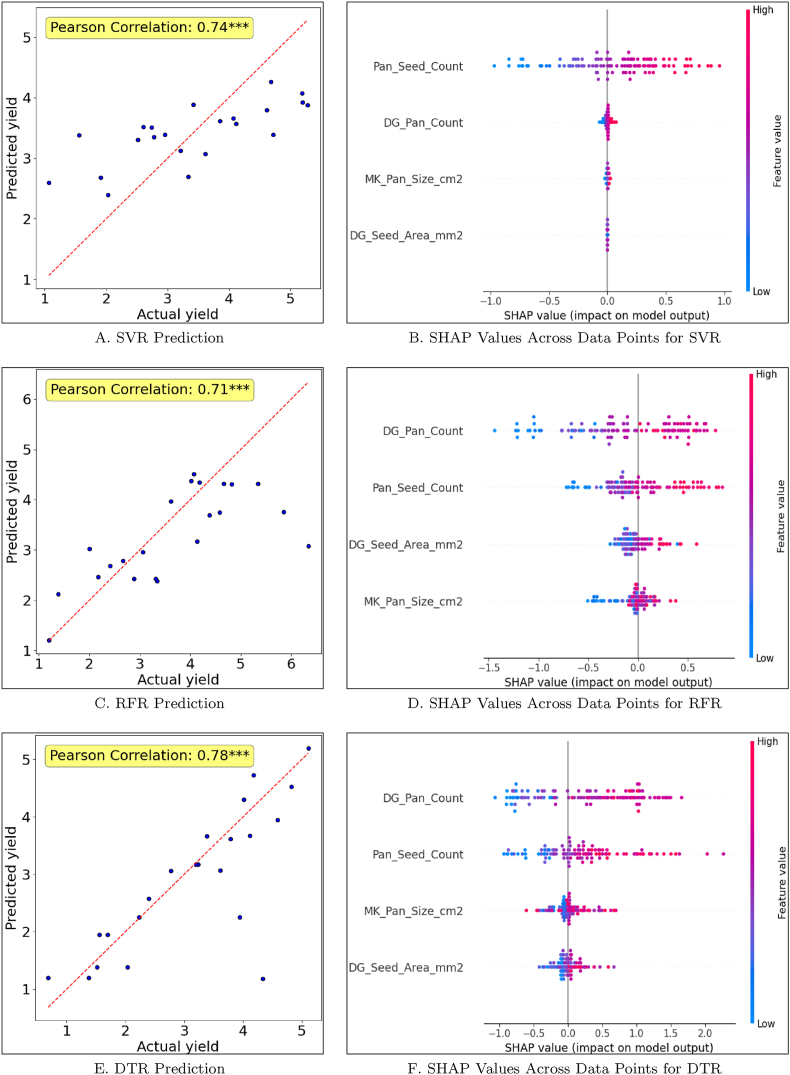
Yield Prediction with Regression models: A. Support Vector Regression (SVR) prediction on the test dataset. B. SHapley Additive exPlanations (SHAP) Values for SVR on the test dataset C. Random Forest Regression (RFR) prediction on the test dataset. D. SHAP Values for RFR on the test dataset. E. Decision Tree Regression (DTR) prediction on the test dataset. F. SHAP values for DTR on the test dataset. For A, C, and E, the y-axis is the ML regression model predicted yield, and the x-axis is the actual harvested yield.

(a) A. SVR Prediction (b) B. SHAP Values Across Data Points for SVR (c) C. RFR Prediction (d) D. SHAP Values Across Data Points for RFR (e) E. DTR Prediction (f) F. SHAP Values Across Data Points for DTR.

The SHAP analysis allows for both local interpretation for specific data points as well as global interpretation at a dataset level. For example, when interpreting the prediction for an individual data point (randomly selected for illustration purposes here), the SHAP analysis revealed that panicle seed count (*Pan_Seed_Count*) was the most influential feature for both SVR and DTR (Fig. S8). For the RFR model, panicle count (*DG_Pan_Count*) coupled with panicle seed count, contributed the most significantly to the prediction of RFR. When assessing SHAP values globally across all test data points, panicle seed count consistently emerged as the pre-dominant predictor for SVR, while other features exhibited minimal influence. In contrast, both digital panicle count and panicle seed count played crucial roles in driving predictions for DTR and RFR models across the entire test dataset (Fig. 9 B, D, F).

## 4 Discussion

The UAV flights were manually operated in this study, even though automated flights were possible with the standard equipment. Developing the automated flight would have been more time-intensive, without adding value to the collection protocol for this type of work. We then applied DL learning open-source algorithms in the UAS-derived field images and manually collected lab images to extract yield-predictive traits in the following successive studies:

### 4.1 Field panicle detection

The sorghum panicle number or density is a critical determinant of yield [11,45]. In this study, we assessed the performance of YOLOv7, YOLOv8, YOLOv9, and Faster R-CNN models using various metrics to understand their effectiveness in sorghum panicle detection and localization. The results indicated a clear distinction in performance among the models in detecting sorghum panicles under different training conditions and view angles. While the Detectron2 based Faster R-CNN model showed reasonable performance, YOLO algorithms outperformed specifically when trained with ND and All view angle images.

Among the YOLO series, YOLOv7 and YOLOv8 exhibited reliable object detection across several metrics and view angles when models were evaluated in the test dataset (Table 1). YOLOv7 achieved the highest precision of 0.90 (AP@0.50-0.95) when trained in the ND view images with more distinct panicles, indicating superior feature extraction and localization capabilities in this set of images. The results highlight the potential of YOLO models, particularly YOLOv7, for precision panicle detection in field conditions. While YOLOv8 delivered competitive results, its performance was slightly lower than YOLOv9. Overall, YOLO algorithms reflected high accuracy and robustness in detecting field panicles, showcasing promise for precise object detection-related field phenotyping.

The superior performance of models trained on the ND view images suggested the importance of clearly visible datasets. ND view images provided consistent panicle orientations with minimal occlusions or overlapping as the camera imaged from the top with a 90° angle, enabling more effective feature extraction and learning. In contrast, FN and FS viewed images introduced challenges such as increased occlusions, non-uniform lighting, and shadow effects, complicating object localization and classification. Various panicle orientations in All view datasets addressed these challenges and created a robust training set. Training with such diversified datasets improves the robustness of the model, as evidenced by the consistent performance of multiple YOLO versions (Table 1). This reinforces the importance of varied data to counteract the impact of field-specific imaging conditions.

Faster R-CNN detected sorghum panicles less effectively, particularly in FN and FS views, compared to YOLO models. This reduced performance could be attributed to the two-stage approach of the algorithm, which could be less efficient in operating dynamic complexities of field imagery compared to the one-stage architecture of YOLO. Consistent with our findings, other authors reported the sensitivity of Faster R-CNN algorithms to background noise and struggle with detecting small objects [46,47].

The best weights of the respective fine-tuned models were used to detect panicles in the UAS-derived field imagery of the experimental plots. The strong correlation between ground truth and predicted panicle counts (Fig. 6) further signifies the effectiveness of the YOLO models in accurately quantifying panicle numbers from field images. This is critical for high-throughput phenotyping applications, where accurate panicle counts are instrumental for various evaluations. When trained on All view images, YOLOv7 achieved the highest correlation (0.86∗∗∗) with the ground truth panicle counts, indicating that a diverse dataset enhances the ability of the model to detect field panicles well. YOLOv8 followed closely with a correlation of 0.76∗∗∗. Although diverse training data improve robustness under varied conditions, it can dilute the ability of the model to optimize for specific scenarios.

Interestingly, YOLOv8 outperformed YOLOv7, achieving a correlation of 0.84∗∗∗ compared to 0.77∗∗∗ for YOLOv7 when the trained model weight on ND view images was used to make inferences on ND view images. This suggests that YOLOv8 may better capture specific spatial features or patterns unique to ND view images. These results imply that training data tailored to ND view angle can optimize model performance for that view, highlighting the importance of view-specific training in field-based phenotyping tasks in certain instances. In brief, YOLOv7 and YOLOv8 consistently demonstrated high correlations in All view and ND view angle training scenarios. This suggests their reliability and adaptability in handling variable field image complexities, including variable view angles and backgrounds.

We computed MAE, NE, and RMSE to evaluate the performance of the model in detecting sorghum panicles. Based on these metrics, slightly different results were obtained; YOLOv8 demonstrated superior performance with the lowest MAE, NE, and RMSE values in recognizing field panicles when evaluated with ground truth data in both instances (fine-tuned weight using All view images and ND view images). This suggests that YOLOv8 is better equipped to understand the pattern, generalize, and make accurate predictions, especially for ND view images.

YOLOv7 also showed competitive performance, particularly in All view and ND inference scenarios, as indicated by its MAE, NE, and RMSE. However, its accuracy slightly declined when trained and evaluated exclusively on ND view images (Table S2).

The performance of YOLOv7, YOLOv8, and YOLOv9 models in detecting panicles has also been evaluated based on F1 scores and Precision-Recall Curve. The F1 scores for YOLOv7, YOLOv8, and YOLOv9 are similar (0.89–0.90). The Precision-Recall Curves illustrated the trade-off between precision and recall at different classification thresholds. The Precision-Recall Curve demonstrated mAP scores of 0.93, 0.92, and 0.94 for YOLOv7, YOLOv8, and YOLOv9, respectively, suggesting the comparative strengths of the models in detecting panicles. Guo et al. [48] conducted a similar study using a two-step machine-based image processing to detect sorghum panicles from UAS imagery. Their method achieved a precision of 0.87 and a recall of 0.98 for detecting sorghum heads. Using a geolocation-based plot segmentation method, they reported precision and recall values of 0.82 and 0.98 for panicle detection. Since the primary goal of this research was yield forecasting through the extraction of yield-attributing features, we prioritized models that could handle dense object detection effectively. YOLOv10, YOLOv11 were not included in our study as it imposes a maximum detection limit of 300 objects per image [49], which is inadequate for accurately detecting a large number of seeds in sorghum panicles. Also, YOLOv11 was released after we completed our model training. Also, Jin [50] reported that YOLOv8 outperformed YOLOv11 for small object detection, further supporting our decision.

### 4.2 Panicle area

Sorghum panicle area, defined by length and width, is crucial for crop yield determination as it is a determinant for the number of seeds in a certain panicle. However, accurately measuring panicle area is challenging due to irregular shapes—many panicles are not straight and may exhibit twisted, curved, or tapered forms with narrow tips or bases. Despite these complexities, panicle area remains a meaningful yield-related trait as the area gives a good indication regarding the number of seeds that the panicle could hold. This experiment demonstrates the potential of digital image analysis for estimating sorghum panicle area, showing a strong correlation (r = 0.79∗∗∗) between SAM 2 mask segmentation based prediction from lab-captured panicle images and ground truth panicle measurements. This high agreement highlights the reliability of deep learning based segmentation (SAM 2) prompted with bounding boxes obtained from a fine-tuned model (YOLOv8) for accurate phenotypic trait estimation.

However, when panicle area was predicted using only the fine-tuned model, accuracy declined (r = 0.59∗∗∗), suggesting that while the model performs reasonably well, it is not yet comparable to mask segmentation. Panicle area through LabelMe annotation offers considerable accuracy (r = 71∗∗∗) but may not be an option for scaling large breeding trials due to time demanding manual labeling. The performance further dropped (r = 0.37∗∗∗) when a fine-tuned model from FN datasets was used to predict panicle area in pixels compared to physical measurements (cm^2^), indicating challenges in translating pixel-based predictions to real-world units. This could reflect issues in scaling, perspective distortion, or conversion factors, especially when working with image-derived data. Panicle area estimation in the lab images was promising but time demanding, as it is associated with manual imaging and annotations.

The findings suggest that this approach can be extended to panicle area extraction from UAS-derived field imagery. However, field conditions introduced significant panicle area and structure variability, making it challenging to assess predicted panicle dimensions accurately against a reliable ground truth.

### 4.3 Seed numbers

The number of grains in a panicle is a critical trait governing sorghum yield [10,51]. Accurately counting seeds within a panicle is challenging due to seed occlusion and the three-dimensional structure of the panicle, which limits visibility in two-dimensional (2D) images. The typical imaging approach can cover only the front and back views, omitting the side views. Furthermore, sorghum panicles exhibit significant variation in size, shape, and compactness, concealing internal grains that are not visible externally [52]. These factors contribute to the complexity of achieving reliable seed counts from the sorghum panicles using standard imaging methods.

We implemented a dual-view approach to address some of the limitations in counting seeds from panicles by predicting seed counts from both the front and back views of the imaged panicle using a fine-tuned YOLOv8 model. Summing these predictions resulted in seed counts with a correlation coefficient of 0.73∗∗∗, compared to ground truth counts. While this method achieved reasonable accuracy for visible seeds, it failed to detect occluded seeds, leading to an underestimation. The observed variation in seed counts was significantly influenced by the shape and compactness of the panicles, which affected seed visibility.

We applied regression-based counting to the panicle images to address concealed seeds. However, contrary to the findings of Bakshi et al. [38], our regression-based approach showed a slightly weaker correlation with ground truth counts.

We hypothesized that images of spread seeds from threshed panicles, offering improved seed visibility, would enable the fine-tuned model to recognize seeds more efficiently. The predictions on spread seed images of harvested panicles yielded a strong correlation of 0.94∗∗∗ with ground truth counts, consistent with our hypothesis. Despite the improved accuracy, this method is time-demanding and requires additional pre-processing, which limits the scalability of large datasets. While developing a non-destructive seed-counting method using field-based UAS imagery remains a desirable goal, the inadequate resolution of current UAS imagery prevented seed counting in this study.

### 4.4 Seed area

The developed graphical interface, with visualization features, can be used for quick and efficient measurement and calculation of seed dimensions and areas, provided adequate pixel resolution is available. This platform lays the foundation for capacity building for extensive and rigorous phenotypic evaluation of small objects like sorghum seeds.

Sorghum seeds are elliptic in shape, and only the dorsal portion of the kernels is exposed when attached to the panicle. This partial exposure limits the ability of image-based methods to capture the comprehensive appearance of the grains. The lack of proper exposure of kernels in the panicles is a key limiting factor in achieving seed area measurement directly from the panicle.

We obtained a correlation of 0.25∗∗∗ from the spread images despite extracting the digital seed area from one set of randomly selected seeds while using another set from the same pool for ground truth seed measurement. This platform serves as a foundation for future advancements in phenotypic evaluation of seed areas.

### 4.5 Yield forecasting

To predict yield (ton/ha), we used digitally obtained yield parameters as independent variables. Based on PCA and mutual correlation among the extracted features, we identified and used four yield-predictive features in the predictive ML regression model to forecast sorghum yield.

Machine learning regression algorithms are prevalent in predictions and also for predictive modeling in agriculture. Among the most widely used machine learning algorithms are SVR, DTR, and RFR due to their flexibility and predictive accuracy. Blockeel et al. [53], upon studying the role of decision tree regressions over four decades, highlighted the importance of tree-based models to specific datasets for optimal performance. In this research, we applied SVR, RFR, and DTR to model yield prediction. DTR emerged as the most reliable model, achieving the highest correlation between predicted and observed yields (r = 0.78∗∗∗), as well as the lowest error metrics: MAE = 0.49 ton/ha, NE = 0.16, and RMSE = 0.84 ton/ha. These results suggest that DTR was particularly effective in capturing the underlying structure of the yield response variable. Although DTR models are known to be susceptible to overfitting—especially without appropriate pruning or control of tree depth [24,54], the performance observed in our study indicates a well-generalized fit to this data.

SVR and RFR also showed promising results, though with slightly lower predictive accuracy. SVR yielded higher error terms (MAE = 1.35 ton/ha, RMSE = 1.66 ton/ha), possibly reflecting sensitivity to kernel choice and the challenges of tuning in high-dimensional, nonlinear spaces [55]. RFR, on the other hand, showed moderate performance (MAE = 0.72 ton/ha, RMSE = 1.02 ton/ha), which aligns with its design: as an ensemble method, it reduces overfitting by averaging across multiple decision trees and is well-suited to datasets with complex interactions [22,56]. Thus, DTR proved to be an accurate and reliable model for yield prediction in this trial, with RFR, and SVR also showing promise.

However, Yamparla et al. [57] reported Random Forest as the best yield predictor with an accuracy of 95 % after training from temperature, rainfall, yield, and pesticide data. In the same manner, Savaliya et al. [58] demonstrated that a hybrid model combining K-Means clustering with Random Forest achieved a prediction accuracy of 99.77 % using weather and soil parameters. However, it is important to note that model performance is highly dependent on the dataset characteristics. For instance, an innovative Gradient Boosting approach reported to be outperformed Random Forest on a smaller dataset (98.7 % vs. 86.4 %) [59]. The SHAP analysis revealed the key role of panicle seed count in predicting yield across the machine learning models. This suggests that seed count within panicles is a primary determining feature of yield prediction, aligns with its biological relevance and our favorite hypothesis. The additional importance of digital panicle count in the Decision Tree Regressor and Random Forest Regressor models indicates that these tree-based methods capture complementary structural information related to panicle architecture, which Support Vector Regressor alone does not emphasize. Together, these findings demonstrate how SHAP confirms the central influence of panicle seed count but also uncovers model-specific feature dependencies, providing deeper insight into how different algorithms weigh biological traits in yield prediction.

### 4.6 Limitations

The variable shape and posture of field panicles [5], complex background, occlusion of seeds in the panicle [38], and insufficient pixel resolutions in the UAS-derived images were some of the key limitations that hindered the precise extraction of yield attributes. Despite these challenges, we generated substantial datasets and object detection protocols to extract yield-predictive traits with greater accuracy. We could not fly a drone (UAV) lower than 6m to get higher-resolution images because that would have disturbed the sorghum plants due to the turbulence created by the spinning propellers of the UAV. The UAV imagery available for this study lacked the spatial resolution and ground-truth measurement necessary to reliably extract panicle features. For this reason, panicle traits from UAV imagery were not included in the present manuscript. We are currently conducting a follow-up study in which ground-truth panicle measurements are being collected in parallel with UAV flights. This will allow us to incorporate UAV-derived field panicle features in future efforts. We are also working on improving spead seed image collection protocol. We would need a longer focal lens and/or a higher megapixel sensor to get higher-resolution images. When trained on relatively small datasets, DTR demonstrated slightly greater precision compared to other regression models. However, a more robust dataset is required to effectively evaluate the predictive performance of the predictive regression analytics. Enhancing data collection methods with more precise sensors can enable higher-resolution imaging, addressing these constraints. Additionally, camera and sensor technology advancements, standardized viewpoints, optimized lighting conditions, and improved scaling methods can further enhance the accuracy of extracting yield-predictive features or traits from the collected imagery and improve yield prediction.

## 5 Conclusion

Advancements in AI, DL, and ML hold immense potential in advancing agricultural research [10,11,60]. With advanced computational power and the availability of various ML algorithms, plant breeders can efficiently extract key yield-predictive features, gain rapid insights into genotype performance, simplify, and improve selection processes. For example, breeders can eliminate a substantial proportion of less promising candidates early in the breeding cycle, such as the bottom 70 %, narrowing their focus to thoroughly evaluate top-performing genotypes. Such a targeted approach enhances breeding decisions in identifying suitable genotypes with desirable traits and accelerates progress toward desirable goals [61].

Automated yield prediction is a pressing demand to keep pace with the need to reach breeding goals rapidly. The findings from our panicle detection study highlight the performance of the YOLOv8 and YOLOv7 models over Faster R-CNN in terms of precision and localization accuracy. However, YOLOv8 was deemed more accurate in the large field plot images in localizing panicles. The measured panicle area from the field was difficult due to the lack of proper resolution in capturing the panicle area variability. The feature extraction from lab-captured images provided greater accuracy, when using SAM 2 mask segmentation. These results suggest strong potential for applying segmentation-based approaches to improve field phenotyping. We aim to leverage this methodology for field-scale panicle feature extraction in future endeavor. Deep learning platforms also exhibited promise in sorghum seed counting and seed area estimation.

Accurate and automated sorghum yield forecasting is cardinal for breeding selections, farm management, and resource allocation. This research aims to improve traditional practices by integrating DL frameworks with UAS-derived panicle imagery to create an efficient pipeline for extracting yield attributes. This methodology offers early yield prediction by leveraging the extracted yield attributes into ML regression algorithms. Integrating SVR, RFR, and DTR, we gained prediction accuracies of 0.74, 0.71, and 0.78 with the actual yield and the SHAP analysis highlights the most influential feature is panicle seed count. The findings demonstrate the potential of DL driven features to forecast yield and thus could enhance crop management, breeding, and policy decisions, accelerating high-yielding cultivar development. Our research also presented a pipeline for extracting yield-predictive features for forecasting sorghum yield by integrating DL frameworks with UAS and manual imagery, contributing to the digitization of sorghum and other breeding and research programs.

## Author contributions

**Md. Abdullah Al Bari**: Conceptualization; methodology, image preparation, data curation, analyses execution, predictive model, SHAP analyses; writing – original draft, review, editing. **Aliva Bakshi**: Faster R-CNN/Detectron2, YOLO models, seed counting analysis, review, editing. **Jahid Chowdhury Choton**: SAM 2 based annotation tool, YOLO models, panicle count predictions, panicle size, seed area analysis, predictive model, SHAP analysis, programming support, review, editing. **Swaraj Pramanik**: Faster R-CNN/Detectron2, YOLO models, seed counting analysis, review, editing. **Trevor D. Witt**: UAS image acquisition, review, editing. **Doina Caragea**: Conceptualization, resources, overseeing analyses, review, editing. **Scott Bean**: Ground truth seed area measurement, review, editing. **S.V. Krishna Jagadish**: Funding acquisition, resources, review, editing. **Terry Felderhoff**: Conceptualization, resources, overseeing analyses, review, editing.

## Funding

This project was funded by the United Sorghum Checkoff Program, Award # CI005-22.

## Data availability

The scripts, fine-tuned models, datasets, and sample images pertinent to this manuscript are available on GitHub at https://github.com/mbari78/DL_for_Sorghum_Yield_Prediction.git.

## Conflict of interest

All the authors hereby declare no conflict of interest.

## Figures and Tables

**figs1:**
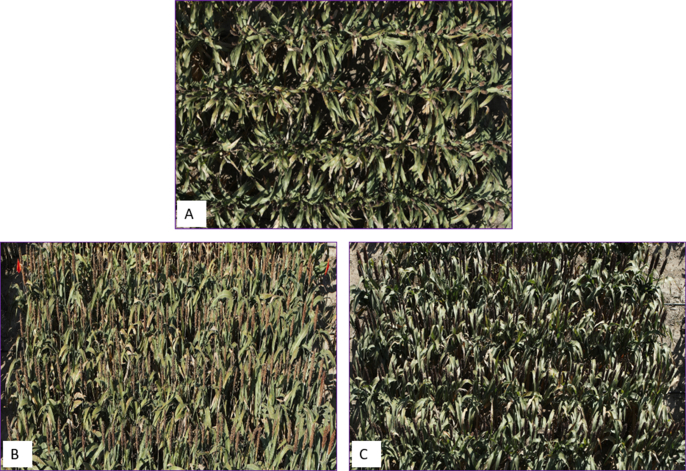


**figs2:**
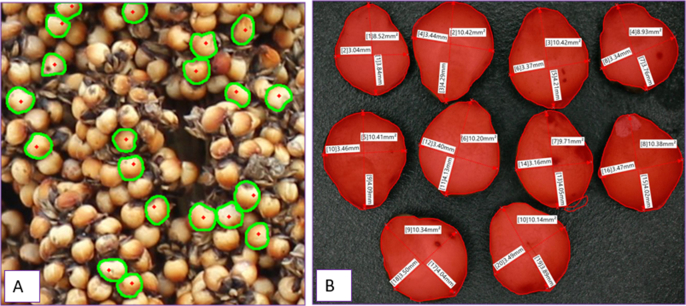


**figs3:**
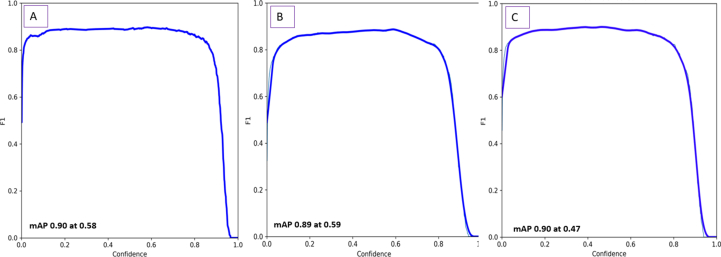


**figs4:**
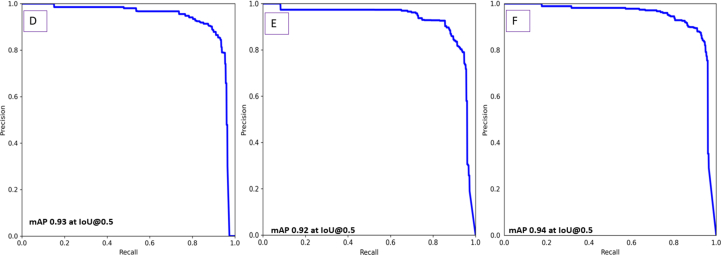


**figs5:**
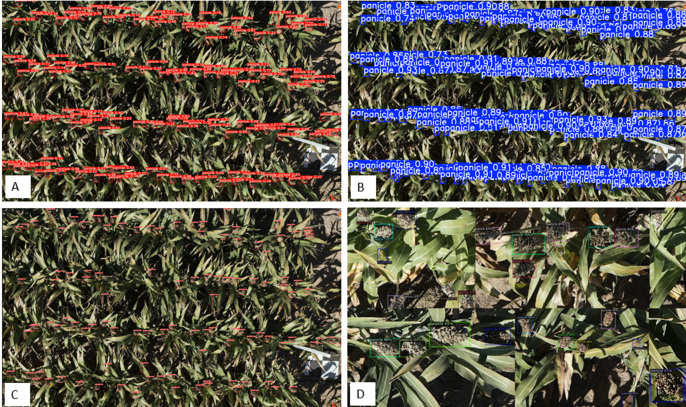


**figs6:**
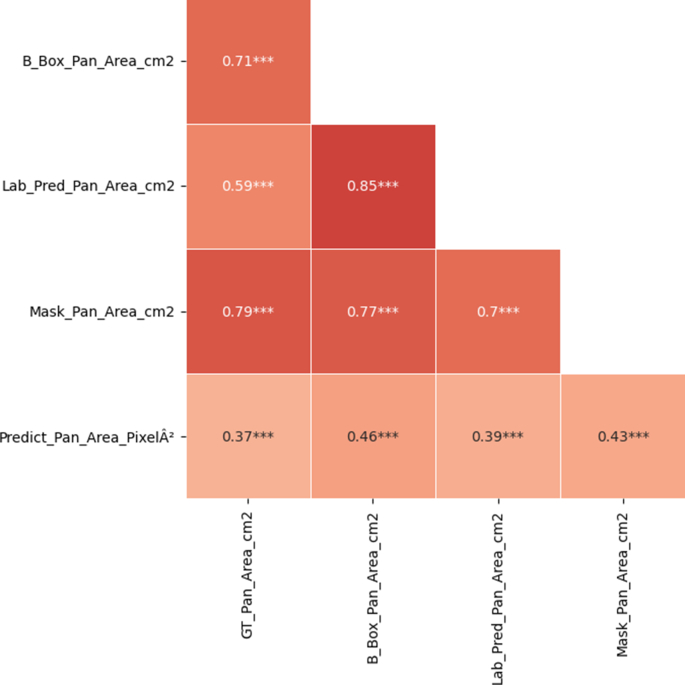


**figs7:**
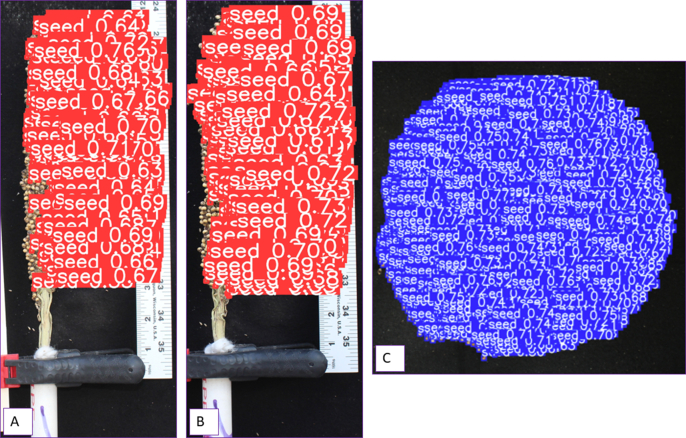


**figs8:**
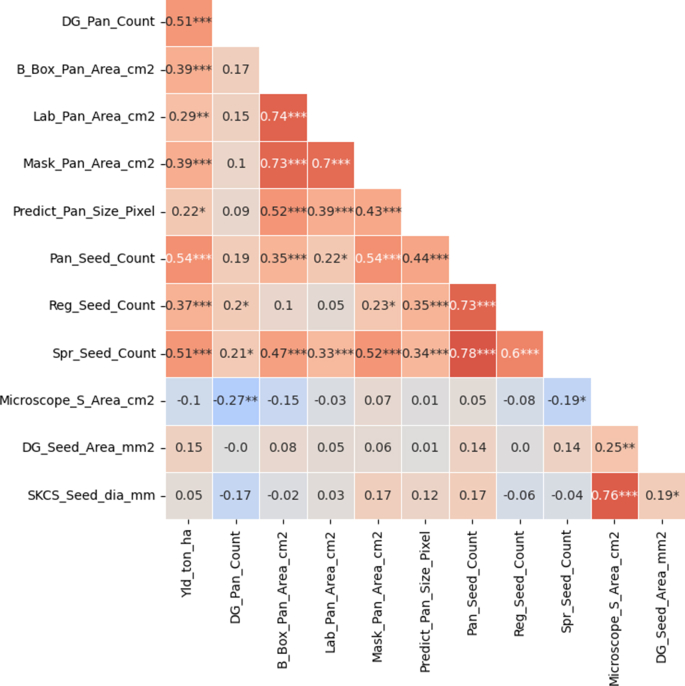


**figs9:**
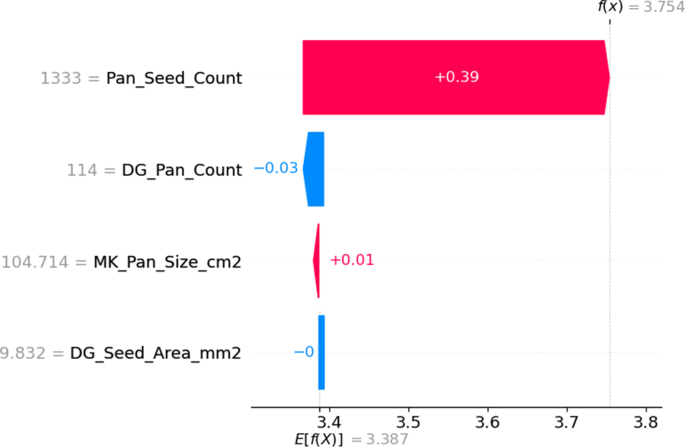


**figs10:**
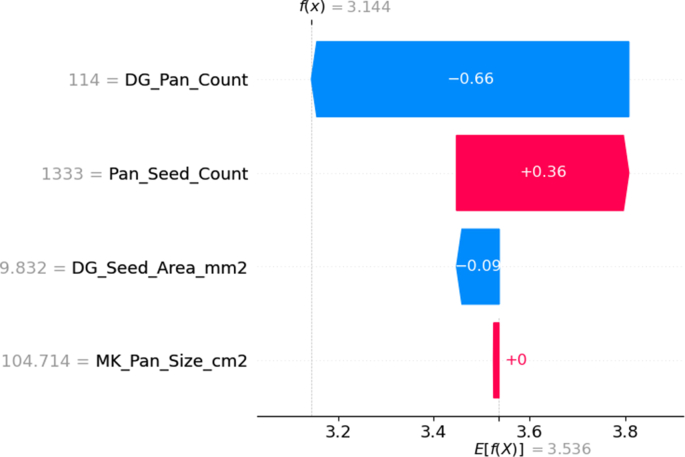


**figs11:**