# Supplementary material for: Deep learning for sorghum yield forecasting using uncrewed aerial systems and lab-derived imagery
Source: Plant Phenomics. 2025 Dec 12;8(1):100133. doi: 10.1016/j.plaphe.2025.100133 (PMC13109310; doi:10.1016/j.plaphe.2025.100133)
Supplement: Multimedia component 1 [file mmc1.docx]

Table S1. Generated images for panicle detection from different view angles

| **View Angle** | **Initial Images** | **Augmented Images** | **Training** | **Validation** | **Testing** |
| --- | --- | --- | --- | --- | --- |
| ND | 303 | 841 | 807 | 17 | 17 |
| FN | 200 | 614 | 594 | 11 | 9 |
| FS | 195 | 547 | 528 | 10 | 9 |
| All Views | 581 | 1627 | 1569 | 29 | 29 |

* ND: Nadir view, FN: Facing North, FS: Facing South.

* All Views include combined images from multiple view angles.

Table S2. Comparative accuracy of Deep Learning models in detecting panicles using ND view imagery

| **Statistics** | **YOLOv7** | **YOLOv8** | **YOLOv9** | **Faster R-CNN** |
| --- | --- | --- | --- | --- |
| **Trained with All view angle images** | | |  |  |
| MAE | 18.97 | **16.42** | 48.39 | 47.58 |
| NE | 0.13 | **0.11** | 0.32 | 0.31 |
| MSE | 537 | 659 | 3085 | 3338 |
| RMSE | 23.17 | 25.67 | 55.54 | 57.78 |
| **Trained with ND view angle images** | | |  |  |
| MAE | 26.89 | **14.33** | 72.94 | 23.75 |
| NE | 0.18 | **0.09** | 0.48 | 0.16 |
| MSE | 1213 | 428 | 6513 | 806 |
| RMSE | 34.83 | 20.68 | 80.7 | 28.39 |
| *DL: Deep Learning; MAE: Mean Absolute Error; MSE: Mean Square Error; | | | | |
| NE: Normalized Error; RMSE: Root Mean Square Error. | | | |  |
| ***All view angle images:** Images combined from multiple view angles. | | | |  |
| ***ND view images:** Nadir view (top-down) images | | |  |  |
