# Supplementary material for: Deep learning for sorghum yield forecasting using uncrewed aerial systems and lab-derived imagery
Source: Plant Phenomics. 2025 Dec 12;8(1):100133. doi: 10.1016/j.plaphe.2025.100133 (PMC13109310; doi:10.1016/j.plaphe.2025.100133)
Supplement: Multimedia component 2 [file mmc2.docx]

| Table S3. Statistics related to the prediction accuracy compared with the respective ground truth observation | | | | | | |  |  |
| --- | --- | --- | --- | --- | --- | --- | --- | --- |
| **Statistics** | **Panicel Area** | | | **Seed Counts** | | | **Seed Area** | |
|  | **Mask_Pan** | **Lab_Pred** | **B_Box_Pan** | **Pan_Count** | **Reg_Count** | **Spr_Count** | **Digital_Area** | **SKCS_Dia** |
| MAE | 54.56 | 46.31 | 38.54 | 468.8 | 449.76 | 172.11 | 2.02 | 8.17 |
| NE | 0.37 | 0.32 | 0.26 | 0.31 | 0.3 | 0.11 | 0.19 | 0.75 |
| MSE | 3590 | 5233 | 3308 | 382514 | 308236 | 57579 | 7.35 | 68.36 |
| RMSE | 59.92 | 72.33 | 57.52 | 618.48 | 555.19 | 239.95 | 2.71 | 8.27 |
| *MAE: Mean Absolute Error; NE: Normalized Error; MSE: Mean Square Error; RMSE: Root Mean Square Error. | | | | | | |  |  |
| ***B_Box:** LabelMe-derived bounding box–based panicle area estimation. | | | | |  |  |  |  |
| ***Lab_Pred:** Lab-image trained bounding box–based panicle area estimation. | | | | |  |  |  |  |
| ***Mask_Pred:** SAM 2–based mask segmentation using predicted bounding boxes from lab-image trained model. | | | | | | |  |  |
| ***Pan_Count, Spr_Seed, Reg_Count:** Seeds on the panicles, seeds using regression, and seeds on the spread images. | | | | | | |  |  |
| ***Digital_Area, SKCS_Dia:** Metrics related to seed area measurement. | | | | |  |  |  |  |
