# Supplementary material for: Deep learning for sorghum yield forecasting using uncrewed aerial systems and lab-derived imagery
Source: Plant Phenomics. 2025 Dec 12;8(1):100133. doi: 10.1016/j.plaphe.2025.100133 (PMC13109310; doi:10.1016/j.plaphe.2025.100133)
Supplement: Multimedia component 3 [file mmc3.docx]

| Table S4. Statistics related to the yield prediction accuracy of ML regression models | | | |  |
| --- | --- | --- | --- | --- |
| **Statistics** | **Support Vector Regression** | **Random Forest Regression** | **Decision Tree Regression** |  |
| MAE | 1.35 | 0.72 | 0.49 |  |
| NE | 0.39 | 0.2 | 0.16 |  |
| MSE | 2.75 | 1.03 | 0.7 |  |
| RMSE | 1.66 | 1.02 | 0.84 |  |
| * ML: Machine Learning; MAE: Mean Absolute Error; MSE: Mean Square Error; | | | |  |
| NE: Normalized Error; RMSE: Root Mean Square Error. | | |  |  |
| * Models evaluated: Support Vector Regression, Random Forest Regression, and Decision Tree Regression. | | | | |

Table S5. Principle component analysis (PCA) showing the contribution of yield-predictive features to yield

| **Features** | **Contributing Variance** |  |
| --- | --- | --- |
| **Mask Panicle Area in cm²** | 0.415 |  |
| **Spread Seed Count** | 0.407 |  |
| **Panicle Seed Count** | 0.406 |  |
| **Bounding Box Panicle Area in cm²** | 0.393 |  |
| **Lab Panicle Area in cm²** | 0.341 |  |
| **Predict Panicle Area in Pixel** | 0.335 |  |
| **Regression Seed Count** | 0.239 |  |
| **Digital Panicle Count** | 0.146 |  |
| **Digital Seed Area mm²** | 0.065 |  |
| Microscope Seed Area mm² | 0.04 |  |
| SKCS Seed diameter mm | 0.036 |  |
| * Features are ranked by their proportion of variance contribution. | |  |
| *Colors in the original figure indicate correlated trait (Figure S7): magenta – panicle area; | | |
| orange - seed count; olive - seed area |  |  |
| *Features shown in bold (in the original) were included in the ML models for forecasting yield. | | |
